# Planning theory- and evidence-based behavior change interventions: a conceptual review of the intervention mapping protocol

**DOI:** 10.1186/s41155-017-0072-x

**Published:** 2017-10-18

**Authors:** Gerjo Kok, Louk W. H. Peters, Robert A. C. Ruiter

**Affiliations:** Maastricht University, Maastricht, the Netherlands

**Keywords:** Intervention Mapping, Behavior change, Health promotion, Intervention, Theory, Participation, Ecological

## Abstract

This paper discusses the Intervention Mapping (IM) protocol for planning theory- and evidence-based behavior change interventions. IM has been developed in the field of health promotion in 1998 and has mostly been applied in that field, but applications in other fields are emerging. IM can be used for any intervention that involves changing behavior. The paper discusses the protocol and its basic issues and presents in-depth examples of its use in- and outside the health promotion field: Empowerment, return to work, safety interventions, implementation, energy conservation, and academic performance. IM is characterized by three perspectives: a social ecological approach, participation of all stakeholders, and the use of theories and evidence. Through a series of six iterative steps - from needs assessment to implementation and evaluation - which are each broken down into specific tasks, correct application of the protocol is meant to produce behavior change interventions that fit into the local context and that have the best chances of effectiveness. IM helps intervention planners develop the best possible interventions targeting health behaviors, but also targeting behaviors related to other societal issues, such as environmental concerns, safety and discrimination.

## Introduction

In the following we will present an overview of Intervention Mapping (Bartholomew Eldredge et al., 2016), a protocol for planning theory-based and evidence-based behavior change programs. Intervention Mapping (IM) is known as a planning protocol for health promotion, but the protocol can be applied to any situation in which behavior change is desirable. We will describe the protocol and its basic issues and present in-depth examples of its use in- and outside the health promotion field: Empowerment, return to work, safety interventions, implementation, energy conservation, and academic performance.

## Review

### Intervention Mapping: Perspectives

IM is characterized by three perspectives: (1) *a social ecological approach* that recognizes that behavior is a function of individuals and of the physical, social and organizational environments in which individuals live; (2) *participation of all stakeholders* to ensure relevance, acceptability, contextual and cultural appropriateness, and implementability; and (3) *the use of theories and evidence*.

**Fig. 1:**
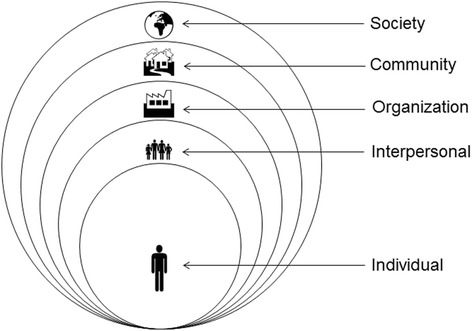
The Social Ecological Approach

In *the social ecological approach*, health is viewed as a function of individuals and of the environments in which individuals live including family, social networks, organizations, communities, and societies (Crosby et al., 2011; Hawe et al., 2009; Kok et al., 2008; Kok et al., 2012; Marmot, 2000; McLeroy, 2006; Trochim et al., 2006;), see Fig. 1. Interventions are events in systems; other factors within a system can reinforce or dampen the influence of an intervention on the specific behavior or environmental change being targeted. In IM we identify agents (decision makers or actors) at each ecological level: interpersonal (e.g., parents), organizational (e.g., managers), community (e.g., newspaper editors), or societal (e.g., legislators). Interventions at the various levels focus on agents (individuals or groups, such as boards or committees) in positions to exercise control over aspects of the targeted environmental condition. The picture that emerges is a complex web or system of causation as well as a rich context for intervention (Bartholomew Eldredge et al., 2016, p. 8–10).

All health promotion program development, implementation, and evaluation should be based on *participation of all stakeholders* (Freire, 1973; Israel et al., 2008; Minkler, 2005; Wallerstein & Duran, 2006; Wallerstein et al., 2015). Inclusive community participation helps to ensure that the program focus reflects concerns for the local community. Broad participation can bring a greater breadth of skills, knowledge, and expertise to a project and can also improve external validity of interventions and evaluation by recognition of the local knowledge of community members and practitioners (Bartholomew Eldredge et al., 2016, p. 10–11).

The *use of theories and evidence* is different in theory-driven versus problem-driven applied behavioral science. Theory-driven applied behavioral science refers to testing a theory in an applied setting, merely to get insight into the validity of the theory. Problem-driven applied behavioral science refers to scientific activities that focus at changing or reducing a practical problem by using a multi-theoretical (social) psychological approach. Although theories are used, the primary focus is on problem solving and the criteria for success are formulated in terms of problem reduction. Problem-driven applied behavioral scientists have to start with a thorough analysis of the practical problem in question, and they have to consider multiple theoretical perspectives to find answers to this problem. The IM protocol suggests the so-called “core-processes”: posing questions, brainstorming what is already known, reviewing findings from the literature on the topic, reviewing theories for additional constructs, assessing new data, and developing a working list of answers (Bartholomew Eldredge et al., 2016, p. 20–23; Buunk & van Vugt, 2013; Ruiter et al., 2012).

Theories can be very useful from a practical point of view. For example, Peters et al., 2009examined effective elements of adolescent health promotion programs in a review of reviews, trying to find elements that were identified to be effective in all domains: one of their conclusions was that theory-based programs produce better effects than non-theory-based programs. Researchers should not restrict themselves to one social-psychological theory, but they should consider theories for all aspects of the problem.

For assessing new data, the program planner needs to combine qualitative and quantitative research (Steckler et al., 1992). For example, Lehmann, et al. (2014) performed focus groups to obtain insight into the determinants influencing health care providers’ intentions to get vaccinated against influenza in Belgium, German, and Dutch hospital settings. They then developed a questionnaire to quantify the results of the focus groups and to gain insight into the relative importance of the determinants that influenced intentions to vaccinate (Lehmann et al., 2015). Qualitative techniques may also be applied afterwards to better understand the meaning of quantitative findings, or in parallel with quantitative methods (Fernandez et al., 2008; Wingood, Hunter-Gamble & DiClemente, 1993; Wittink et al., 2006).

### Intervention Mapping: Steps and tasks

**Fig. 2:**
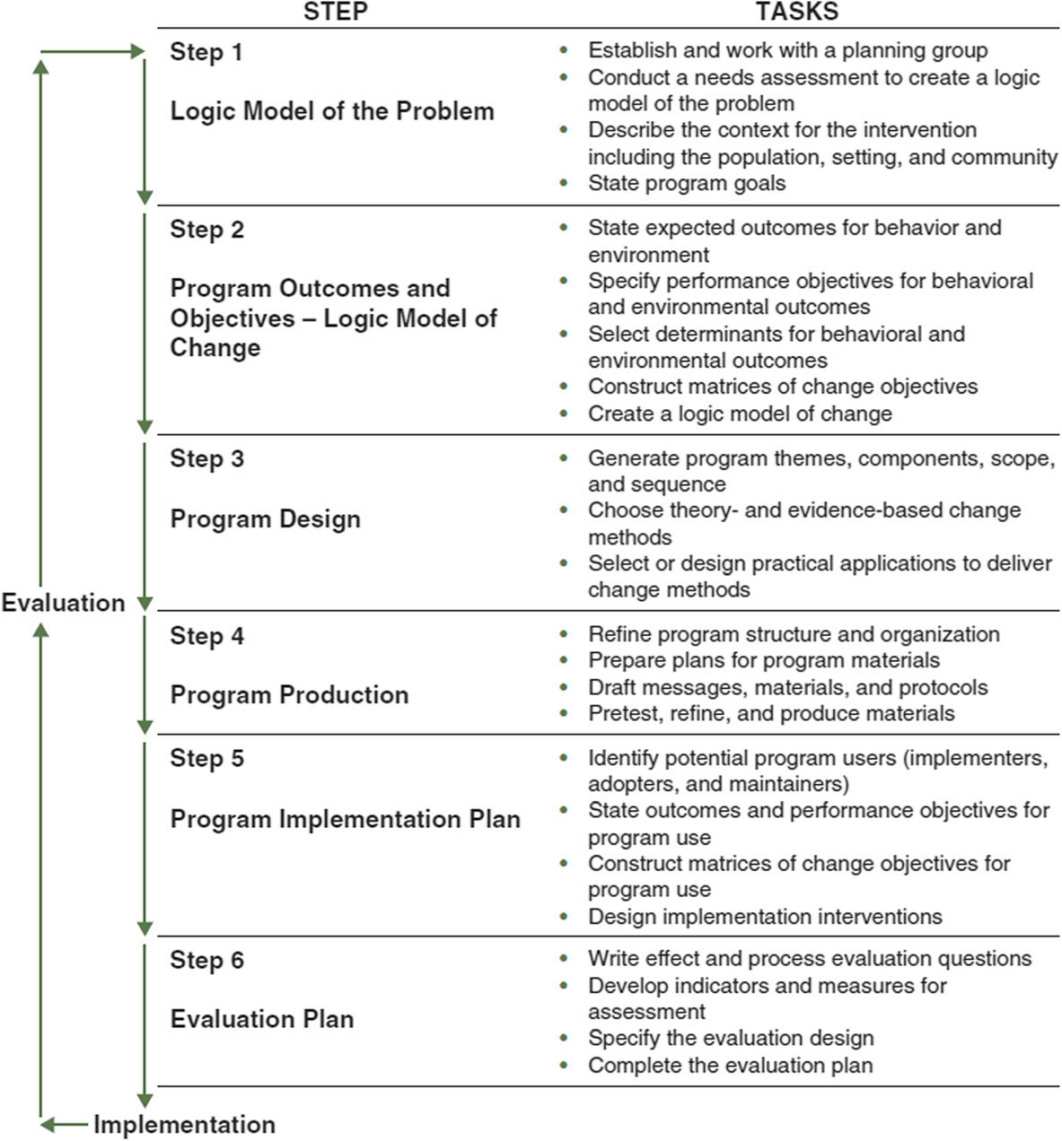
Intervention Mapping Steps and Tasks

IM describes the process of program planning in six steps, with each step comprising several tasks (see Fig. 2, Bartholomew Eldredge et al., 2016, p. 13). Completion of all steps creates a blueprint for designing, implementing and evaluating an intervention based on a foundation of theoretical, empirical and practical information.

In the first step the planner puts together a planning group to assess the problem, the behavioral and environmental causes of the problem, and determinants of behavioral and environmental causes, which are then depicted in *a logic model of the problem*. In addition, it is important to understand the character of the community and its knowledge of the problem and potential solutions. Use of the PRECEDE-PROCEED model is advised in this step (Green & Kreuter, 2005). Finally in this step, the planning group sets goals for intervention, including behavioral and environmental change. Step 2 specifies program outcomes and objectives in *a logic model of change*. Changes in specific desired behaviors of the risk group or environmental agents (performance objectives) and changes in determinants (change objectives) are defined, based upon scientific analyses of (health) problems and problem causing factors. In step 3, a coherent, deliverable intervention is designed. Theory-based intervention methods and practical applications to change (determinants of) behavior are selected, and program themes, components, scope and sequence are generated. Step 4 comprises the actual production of the intervention. Program structure is refined, and messages and materials are drafted, pretested, and produced. In step 5 a program implementation plan is generated. Potential program users are identified, performance objectives and change objectives for program use are specified, and implementation interventions are designed. In step 6 a plan is generated for effect and process evaluations. An evaluation plan is actually begun in the needs assessment and is developed throughout each step. To evaluate the effect of an intervention, researchers analyze the change in health and quality-of-life problems, behavior and environment, and their determinants. Optimally, planners have defined these factors in a measurable way during the preceding steps. Activities for steps 5 and 6 start as early as possible in the planning process.

Although IM is presented as a series of steps, the authors see the planning process as iterative rather than linear. Program planners move back and forth between tasks and steps. The process is also cumulative: each step is based on previous steps, and inattention to a particular step may lead to mistakes and inadequate decisions in the steps following.

### Brief history and purpose of the protocol

IM was first developed and introduced with an article in Health Education & Behavior (Bartholomew et al., 1998) as a result of the collaboration between the School of Public Health at The University of Texas at Houston and the School of Health Sciences at Maastricht University, the Netherlands. In 2001 the first book on IM was published (Bartholomew et al., 2001), followed by later editions in 2006 and 2011, respectively (Bartholomew et al., 2006; Bartholomew et al., 2011). The 4th and newest edition from 2016 was authored by L. Kay Bartholomew Eldredge, Christine M. Markham, Robert A.C. Ruiter, María Fernández, Gerjo Kok, and Guy S. Parcel. The authors represent various disciplines: health promotion, educational science, social psychology, and sociology. IM was also published as a taxonomy of behavior change methods in Health Psychology Reviews (Kok et al., 2016).

IM was developed as a reaction to a lack of comprehensive frameworks for health promotion program development. At that time IM was seen by the IM authors as an extension of the PRECEDE-PROCEED model, as Green and Kreuter (2005) did not provide much detail on the actual program development and the use of theory and evidence in that process (in the first edition of the book, PRECEDE-PROCEED was seen as a preparational step; later it became step 1). In the United Kingdom, the Medical Research Council published a ‘Framework for the Development and Evaluation of RCTs for Complex Interventions to Improve Health’ (MRC, 2000), which has been updated since (Craig et al., 2008), to help researchers and research funders to recognize and adopt appropriate methods. While their focus is somewhat different, the basic ideas are comparable to PRECEED/PROCEED and IM. As mentioned above, IM provides more detail on how to apply theories and evidence from the behavioral sciences to health promotion program planning (Buunk & Van Vugt, 2013).

IM aims to help health promoters develop the best possible intervention. The key words in this protocol are planning, research, and theory. IM provides a vocabulary for intervention planning, procedures for planning activities, and technical assistance with identifying theory-based determinants and methods for change. IM can also help in adapting existing interventions to new populations and settings, and provides a taxonomy of behavior change methods that can be used to code intervention content. In the health promotion field, IM has successfully been applied in various settings, to a wide range of different behaviors and populations.

Although IM is considered a helpful tool to design programs, it is true that it is a complex and time-consuming process, reflecting the difficulty of changing (health) behaviors. IM has been described as complex, elaborate, tiresome, expensive, and time consuming (Côté et al., 2008; Heinen et al., 2006; van Kesteren et al., 2006). Interestingly, despite these criticisms, these authors also concluded that IM helped bring the development of interventions to a higher level (Godin et al., 2007), indicating that advantages outweighed disadvantages. IM was developed in the health promotion field but it can easily be applied in other fields. We will present some examples from outside the health promotion field further below, such as safety promotion, energy conservation, and academic achievements.

### Three basic highlights

Although the whole model, with all its steps and tasks, is valid and based on solid reasoning, almost 20 years of experience with IM has led us to identify three highlights, or lessons learned, that are of particular relevance for planning interventions using IM: identifying the determinants of the target behavior, taking into account the theoretical parameters while applying behavior change methods, and making sure that the intervention is implemented as planned.

#### Identifying the determinants of the target behavior

Target behaviors for health promotion are many, often described as preventive or risk-reduction behaviors (e.g. smoking cessation), health promoting behaviors (e.g. bicycle helmets, breast cancer screening), or adherence and self-management behaviors (compliance with medical advice). Identifying the relevant determinants, and underlying beliefs to influence, is a crucial step in intervention development (G. Peters, 2014; Crutzen et al., 2017). Influences on behavior exist inside a person, intrapersonal, or outside: environmental conditions. Determinants are not directly observable but can be measured indirectly. Determinants, such as awareness, attitudes, perceived norms and self-efficacy, consist of specific beliefs: thoughts, associations, perceptions, et cetera. Beliefs relate to specific behaviors, and ultimately predict the focal behavior via determinants and intentions. Target behaviors usually comprise a set of preparatory or sub-behaviors, called performance objectives. An example of performance objectives for adolescents using condoms correctly and consistently (target behavior) may be: buy condoms, carry condoms, negotiate condom use with partner, correctly apply condoms, and maintain condom use over time (Schaalma et al., 1996). Environmental conditions at interpersonal, organizational, community and policy levels can be changed by targeting the decision makers or agents.

Methods for behavior change are usually matched to determinants. How to identify which determinants to change in the first place? Ideally, (1) an overview of the existing literature about the determinants and underlying beliefs of the target behavior is supplemented with (2) qualitative elicitation interviews with target population members and possibly key environmental agents. The results of these two steps are (3) quantitatively verified in a survey so that the relative importance of determinants and beliefs for predicting intentions and behavior can be established. G. Peters (2014) provides concrete guidelines for these tasks, resulting in the so-called matrix of change objectives (see Table 1). In this matrix the performance objectives for a target individual (e.g. monitor physical activity) are crossed with the identified determinants (e.g. self-efficacy), resulting in so-called change objectives (e.g. express confidence about being able to monitor). Table 1 gives an example of such a matrix from the *Active plus* project (Van Stralen et al., 2008), an intervention to promote physical activity in people over 50.

**Table 1:** Sample change objectives (combination of performance objectives and determinants) for promoting recreational physical in the over-fifties, the *Active plus* program (van Stralen et al., 2008)

Performance objectives	Determinants		
	*Awareness*	*Attitude*	*Self-efficacy*
1. Older adults monitor their recreational physical activity level	*Older adults describe the purpose of monitoring and reporting their own recreational physical activity*		*Older adults express confidence about being able to monitor and report their own recreational physical activity*
2. Older adults indicate reasons to be physically active as recreation	*Older adults list the personally relevant benefits of being sufficiently physically active*	*Older adults express a positive attitude about being sufficiently physically active*	
3. Older adults identify solutions to take away the barriers to being physically active for recreation	*Older adults describe the situations and barriers that prevent them from being sufficiently physically active*		*Older adults express confidence about being able to take away and to cope with their barriers*

#### Taking into account the theoretical parameters while applying behavior change methods

As the next part of the process, we now need to link the change objectives to theoretical methods and apply those methods correctly in an intervention (Kok, 2014). In IM we distinguish between change methods at the individual level and change methods at the environmental level (see Table 2). At the individual level, a theory-based behavior change method is a general technique or process designed to influence the determinants of behavior. Change methods are used to target determinants such as attitude or self-efficacy (e.g. modeling) that are in turn thought to influence the behavior. Change methods require translation into practical applications taking into account the theory-based parameters. For instance, translating the theoretical method of *modeling* into a practical application requires choices with respect to the type of person who models, how to portray the behavior to be modeled, how to portray the consequences of the behavior, et cetera. In these choices, the following parameters for effective use of modeling should be taken into account: the learner has attention for the model, the learner can remember the model’s action, the learner will identify with the model, the learner observes that the model is reinforced, the learner has sufficient self-efficacy and skills for the action, and the model serves as a coping model instead of a mastery model (Kok et al., 2016).

**Table 2:** A selection of methods, parameters, and examples of applications (Bartholomew Eldredge et al., 2016; Kok et al., 2016)

Methods, theory & definitions	Parameters for use	Examples of applications
Example of basic method at the individual level		
*Modeling:* Providing an appropriate model; being reinforced for the desired action (Social Cognitive Theory, theories of learning; Kazdin, 2012; Kelder et al. 2015)	Attention, remembrance, self-efficacy and skills; reinforcement of the model; identification with model; coping model instead of mastery model.	The health promoter finds a role model from the community or at-risk group who will encourage identification and serve as a coping model: “I tried to quit smoking several times and failed; then I tried… Now I have been off cigarettes for…”
Example of method to change habitual, automatic, and impulsive behavior		
*Cue altering:* Teaching people to change a stimulus that elicits or signals a behavior (Theories of goal directed behavior, theories of automatic, impulsive and habitual behavior; Verplanken & Aarts, 1999; Wood & Neal, 2007)	Existing positive intention.	Dieters change the places they keep snack food in order to prevent taking the snack automatically.
Example of basic method for change of environmental conditions		
*Coercion:* Attempting to control others against their will (Freudenberg & Tsui, 2014; Turner, 2005)	Requires or creates a power difference.	Health promotion activists organize a consumer boycott of a company that sells formula in developing countries.
Example of method to change social support and social networks		
*Developing new social network linkages:* Linking members to new networks by mentor programs, buddy systems, and self-help groups (Theories of social networks and social support; Holt-Lundstad & Uchino, 2015; Valente, 2015)	Willingness of networks to reach out; availability of networks that can provide appropriate support and linkage agents.	Volunteers who are breast cancer survivors are linked to newly diagnosed patients to provide emotional and informational support.

With respect to environmental levels, methods are linked to each level: interpersonal, organizational, community and policy levels. In order to select appropriate methods for changing environmental conditions in a health intervention, the first step is to find out who may be in a position to make the expected change to identify the desired behaviors for the agent. The behavioral scientist can then apply methods to influence the determinants of the agent’s behavior; using methods for individual change as well as methods that are appropriate for that specific level. Table 2 gives examples of theory-based methods at the individual and environmental levels. Again, change methods at the environmental level also require translation into practical applications taking into account the theory-based parameters. For instance, translating the method *developing new social network linkages* into a practical application requires availability of existing networks and willingness to provide support.

Practical applications are specific translations of theory-based methods for practical use. They should be tailored to the intervention population and the context in which the intervention will be conducted, and take the parameters for use into account. The situation commonly encountered in the “real world” of intervention development is that theory-based methods tend to disappear in translation. Translating methods into practical applications demands a sufficient understanding of the theory behind the method, especially the theoretical parameters which determine whether the theoretical process is effective or not. No method is always effective. For example, *goal setting* can be a very effective method, but only when the goal is challenging as well as acceptable for the actor (Latham & Locke, 2007). If the goal for the target group is fixed and is set by the program planner – as opposed to self-set by the target group or negotiated – preliminary research should make sufficiently clear what (level of the) goal will be perceived as acceptable and challenging by the majority of the target group. Without guidance, people from the target group often choose goals outside those parameters. Or, *fear appeals* are only effective when the at-risk population has high (self-) efficacy, and they may actually be counter-effective when efficacy is low (Peters et al., 2013; Ruiter et al., 2014). Table 2 gives examples of parameters for methods.

How are these practical applications integrated into an effective intervention? Essential in the collaboration with creative consultants is mutual respect: respect the creative professional, but also ensure that the creative professional respects the behavioral scientist. Creative consultants are seldom aware of the parameters for effectiveness that apply to methods, and it is the responsibility of the behavioral scientist to make sure that those parameters will stay intact.

IM suggests that in step 3 intervention planners first describe their ideas about the concrete parts of the program: generate program themes, components, scope, and sequence. A program *theme* is a general overarching construct for a program, sometimes organized into sub-themes. Examples of themes include: the *Active Plus* exercise program for the over-fifties (van Stralen et al., 2008), or *Cultivando la Salud*, an intervention to increase cancer screening among low income Hispanic women in the US (Fernández et al., 2005; Fernández et al., 2009). The *scope* refers to the breadth and size of the program, describing what is and what is not in the program. The *sequence* refers to the order in which the elements of a program are delivered across time. Sometimes existing materials may be useful. All materials and products need to be pilot tested. If possible, apply experimental designs in pretests (Whittingham et al., 2009). Table 2 gives examples of methods, parameters, and applications. Table 3 gives an example of program scope and sequence of the Dutch sex education program with the theme: *Long Live Love*.

**Table 3:** Example of program scope and sequence from the *Long Live Love* sex education program for adolescents (Schaalma et al., 1996)

Lessons and Goals	Practical Applications
*Lesson 1:* To Increase knowledge and to change risk perceptions	Inquiry teaching Classroom discussion Exercises to apply the information that is provided (e.g., making an information brochure, completing a quiz, interviewing peers) Teachers lecture and experts give information in print
*Lesson 2:* To change attitudes about condom use and safer sex in general	Classroom discussion on the basis of a homework assignment addressing facts about AIDS and sexually transmitted infection (STI) prevention Role-model stories in print material covering attitudes about safe sex and problems with practicing safe sex Classroom discussion on the basis of statements about practicing safe sex (students respond to statements orally or in writing) “Paper-and-pencil”-subgroup discussion
*Lesson 3:* To strengthen values, social influences, and communication skills regarding the prevention of AIDS and STIs	Homework assignment requires students to respond to situations addressing social pressures (“What would you do when ....”) Classroom discussion subsequent to homework Teacher-delivered information about the process of social influence (didactic approach or inquiry teaching) Peer models discussing safe sex and telling about their attitudes, values, and experiences by means of dramatized videotape and subsequent classroom discussion about videotape modeling
*Lesson 4:* To enhance students’ self-efficacy regarding negotiating and condom use skills	Homework assignment on buying condoms Subsequent classroom discussion about buying experiences Demonstration and practice of condom use on fingers Video-animation of adequate condom use Interactive videotape showing peer models negotiating real-life troublesome situations and subsequent classroom discussion

#### Making sure that the intervention is implemented as planned

Once the intervention has been created, a solid diffusion and implementation process is vital to ensure program success (Knittle, 2014). Remember that thinking about implementation is an ongoing process straight from the beginning of the planning. For example, a school program with 20 lessons might seem necessary but will be very difficult to implement in schools with already overcrowded curricula. Without implementation, the intervention will not have any chance of impact on determinants, behaviors, or health. In IM Step 5 a plan is developed for the systematic implementation of the program. First one needs to develop a linkage system, making sure that program developers are linked with program users in the planning team; if not, add implementers to the planning group. Next, an intervention is developed to promote adoption and implementation of the program by the intended program users. Intervention planners develop interventions to facilitate the implementation of the health promotion intervention with high fidelity - making sure the intervention is implemented as intended - and high completeness - making sure the full intervention is implemented. They develop theory-based interventions to facilitate program adoption by key stakeholders, to support appropriate implementation by program users, and to encourage program institutionalization by considering opportunities for incorporating the program into organizational routines. Thus, interventions are not only required to change individual behavior, but also to facilitate program implementation. Indeed, the same steps involved in intervention development are repeated to anticipate program diffusion and to target program implementers: performance objectves, change objectives, methods, applications and program. Sustainable implementation almost always involves organizational change, for example in a school setting ‘modeling’ by experienced trainers for teachers and ‘facilitation’ with guidelines and protocols for school principals.

### Applications of Intervention Mapping in intervention planning and research

In 2015, we started a database of references and publications about Intervention Mapping, which now contains over 750 titles (accessible via the Intervention Mapping website http://interventionmapping.com/references). Most of these, over 700, are related to intervention projects in which the use of IM is claimed or planned. These publications relate to over 200 intervention projects and include papers about needs assessment, program development, evaluation protocols, effect evaluation, process evaluation, dissemination, and cost-effectiveness. In most cases, use of IM is only mentioned in papers about program development, so identification of other types of papers from the same project relies on backward and forward search and/or contacting authors. So far, we have identified about 100 projects for which there are papers about use of IM in program development as well as papers about program effects. For the future we intend to conduct a meta-analysis in which the use of IM steps and tasks in these projects will be categorized in detail and will be linked to effect size. In the current paper we will describe some applications of IM in practice.

As said above, IM is mainly applied in the health promotion field, the field in which it was developed. Applications in this field cover about all typical and well-known health-related behaviors (diet, physical activity, sexual behavior, smoking, drinking, drug use, and behaviors related to unintentional injury), but are certainly not limited to these. In addition, topics inside and outside the field of health promotion include: adherence to medication or treatment, adherence to professional standards or guidelines, participation in screening or in vaccination, returning to work after illness or disability, stress management, violence prevention, injury prevention and safety promotion, empowerment, professional decision making in medicine, doctor-patient communication, psychiatric treatment, urban regeneration, energy conservation, promotion of students’ interest and competence in science classes, and nationwide dissemination of programs. Although most IM projects are from Europe and North America, use of IM is spreading and, up to now, has been reported for 46 countries scattered across most continents.

#### Empowerment: The teenage mother project

An example from a developing country is the Teenage Mothers Project, a community-based program to improve empowerment and psychological and social well-being of unmarried teenage mothers in rural Uganda (Leerlooijer et al., 2013, 2014). Unplanned teenage motherhood is associated with low self-esteem, depression, stigma, isolation, rejection from families and community members, limited social and financial support, high rates of school dropout and limited career opportunities. A planning group consisting of community leaders, teenage mothers, staff of a community-based organization and a health promotion professional was involved in the six steps of IM. The group decided to intervene among teenage mothers, parents, community decision makers including religious leaders and tribal chiefs, school principals, and members of the community at large.

The behavioral outcomes for unmarried teenage mothers covered improved coping with stigma and motherhood, continuation of education, increased income generation, abstinence or protected sex and advocacy for the rights of unmarried teenage mothers. Environmental outcomes were also drawn up, for example, for parents the outcome was that they support their daughter to continue education. Related performance objectives for parents were to: a) effectively cope with negative norms in society regarding continued education, b) communicate with the school administrator to allow their daughter to return to school, c) pay school fees and other necessities for their daughter to go to school, d) care for the baby when their daughter attends school. For all target groups determinants of performance objectives were identified, such as knowledge and awareness, attitude, perceived social influence, and skills and self-efficacy, based on interviews and focus groups in the needs assessment.

A comprehensive program was developed which included five intervention components: community awareness raising (meetings, performances and goats-giving ceremonies), teenage mother support groups, formal education and income generation (goat rearing), counseling, and advocacy. In each of the components several theoretical methods were used. For instance, the community awareness raising component included the methods entertainment education, persuasive communication, instrumental support and social action. Practical applications of entertainment education were theater plays and songs performed by the teenage mothers to create awareness and knowledge of stigma and its consequences and attitude change among community members. In public goat-giving ceremonies, giving goats (application of instrumental support to generate income) was combined with speeches by community leaders who supported the project (application of persuasive communication to influence community members’ attitude). The parameters for using each method were acknowledged. For instance, parameters for persuasive communication were that messages need to be relevant, not too be discrepant from the receiver’s beliefs, and to include new arguments. The messages in the project focused on giving unmarried teenage mothers a second chance and on helping them to continue their education, thus still acknowledging the community’s deeply rooted disapproval of out-of-wedlock teenage pregnancy. Throughout the entire project, the general method of cultural similarity was applied. The project deliverers originated from the same tribe and communities as the beneficiaries, contributing to a positive reception of the intervention.

Also, interventions were designed to address program adoption and implementation. Adoption started with sensitization of tribal, religious and government decision makers. They were repeatedly exposed to the project’s messages and to interactions with the teenage mothers, resulting in attitude change and supportive behavior towards the mothers, their parents, other leaders and the community at large. Subsequently they were involved as implementers by giving them a leading role in community meetings and activities of support groups. Journalists were regularly invited to meetings and community occasions, encouraging them to report about the project. Trained coordinators (community-based volunteers) were appointed in each community to implement activities. Moreover, applications were first implemented on a small scale and monitored. If applications were adopted and initiated change, the activities were upscaled and incorporated in the intervention.

The program was evaluated with a qualitative evaluation (Leerlooijer et al., 2013). This evaluation indicated that the project contributed to the well-being of unmarried teenage mothers (increased agency, improved coping, continued education, increased income generation) and to a supportive environment (supportive community norms towards the mothers’ position and future opportunities).

#### Returning to work after gynecological surgery

Vonk Noordegraaf et al. (2012) developed an intervention to empower patients in returning to normal activities and work after gynecological surgery in the Netherlands. There is a large discrepancy between expected duration of physical recovery and actual return to work after gynecological surgery. After discharge, detailed convalescence recommendations are mostly not provided and postoperative care is fragmented, poorly coordinated and given only on demand. For patients, this contributes to irrational beliefs and avoidance of resumption of activities, resulting in a prolonged sick leave and unnecessary costs for society.

In the needs assessment, literature research into behavioral and environmental conditions of prolonged sick leave was supplemented with focus group discussions with patients to establish performance objectives for patients, gynecologists, family physicians (FP), occupational physicians (OP) and employers. Performance objectives for the non-patient target groups were mainly to acquaint themselves with recommendations for their patients (gynecologist, FP, OP) or to show involvement and discuss a work-reintegration plan with their employee (employer). Detailed recommendations for resumption of work were developed in a Delphi study by a multidisciplinary expert panel. Performance objectives for patients were to acquaint themselves with information (recommendations, surgery- and care-related issues, telephone numbers), to not extend their sick leave beyond the recommended period on their own initiative, to develop a work-reintegration plan and discuss it with their employer, to identify barriers for a safe return to work, to exchange experiences with other patients and to receive answers to questions and uncertainties. Determinants were identified as the patient’s attitude, social influence, self-efficacy and skills, as well as external barriers and facilitators.

The program that was developed was a tailored Internet application; www.ikherstel.nl (which means ‘I am recovering’). It had two main sections for patients -a central home page and an action list- and a different section for gynecologists, FPs and OPs which included guidelines, casuistry and background information based on the Delphi study. The action list section for patients consisted of various tools for actions: composition of a plan for medically safe work-reintegration, a plan for gradually resuming normal activities, and a survey to identify complications that require additional consultation. The priority of the different actions was based on the date of surgery and information from the gynecologist. The home page section for patients included a video about common pitfalls during the perioperative and reintegration period, recommendations, a forum to contact other patients, FAQ, a glossary, and links to other websites. Various methods were used in the tools, including persuasive communication, self-re-evaluation, modeling, goal-setting, and mobilizing social support; parameters for these methods were acknowledged.

In the test phase, the eHealth program was evaluated favorably by patients, physicians and eHealth specialists, and in result, only minor adjustments were made. Adoption and implementation were anticipated by involving patients and a linkage system (representatives of national medical boards of gynecologists, FPs and Ops and of a patient organization) in all stages of intervention development and evaluation. Health care providers, OPs and eHealth specialists participated in the test phase of program production. Furthermore, use of the intervention by the various target groups was intended to be easy and without any need for support.

The eHealth intervention was evaluated in an RCT among 215 patients from 7 hospitals in the Netherlands (Vonk Noordegraaf et al., 2014). It was compared to a control eHealth website; both websites were offered as a supplement to standard perioperative care. The intervention had favorable effects on time to full sustainable return to work (hazard ratio 1.49; medium duration compared to control group was 39 versus 46 days), pain intensity, and quality of life.

#### Worksite health promotion: Safety interventions in small metal fabrication businesses

Brosseau et al. (2007) and Parker et al. (2009) developed, implemented and evaluated an intervention to lower machine-related hazards and amputations in small metal fabrication businesses in the USA. They compared two interventions; one intervention for business owners only, and one for both business owners and employees. Their planning group consisted of owners, economic development consultants, occupational health and safety professionals, technical school faculty and a union representative. They also had group meetings with employees. Examples of performance objectives for owners were: assess adequacy of shop safety, and become familiar with safety improvements. Examples of performance objectives for employees were: assess machine safety, and participate in health and safety decision making. Identified determinants were: self-efficacy, reinforcement (e.g. incentives), perceived environment (e.g. safety climate), as well as environmental factors, such as social support from coworkers. Intervention components were linked to determinants and targeted the chosen change objectives. An important component was building the skills of a health and safety committee, with at least two employees and one manager with safety responsibility. Machine safety audit and survey results were used to tailor information and skills training for each committee. Other components were role models and peer trainers. Brosseau et al. report that the implementation of their interventions proceeded relatively smoothly. Parker et al. evaluated both interventions and concluded that they both significantly improved the adequacy of machine guarding and of safety programs. Businesses that added health and safety committees, or those that started with the lowest baseline, showed the greatest improvements.

#### Implementation: Dissemination of the JNC 7 guidelines

Bartholomew et al. (2009; Stafford et al., 2010) applied Intervention Mapping in an intervention to promote the dissemination of clinical trial results among USA physician practices; the JNC7 guidelines. The goal was to promote the compliance to these guidelines, i.e. the performance objectives, beyond the standard publications and presentations of trial results. The target population consisted of health care providers who manage hypertension. Determinants, benefits and barriers that were identified included awareness, outcome expectations, skills and self-efficacy, perceived norms and standards of care, as well as pharmaceutical marketing influence. The main strategy in the intervention was the ‘investigator educator’ approach: physicians that educate other physicians, as role models, through small group presentations. The proposed settings were medical practice staff meetings, hospital staff meetings, residency programs, local medical societies and other venues as identified by the educator or project manager. The educator also left cues to action such as newsletters with role model stories, pocket cards with guidelines and tips, and posters to encourage patients to talk with their doctors. A second strategy for reaching health care providers was through their professional associations.

The intervention reached more than 18.500 physicians in 41 states. These participants demonstrated statistically significant positive differences in pretest versus posttest scores. A later effect evaluation showed that the counties with the most project activities showed the highest increase in following the JNC7 guidelines. Bartholomew et al. (2009) conclude that the intervention has potential but it may need greater intensity to facilitate implementation of guidelines in practice.

#### Energy conservation: Fuel saving driving style of mail van drivers

In an example of theory use in program development from the pre-IM era, the first author of this paper was involved in a project targeting the modification of the driving behavior for energy saving (Siero et al., 1989; Kok et al., 2011). This intervention promoted a fuel saving driving style among van-drivers of a mail company. Important energy-wasting behaviors among van-drivers were identified: pressing the gas pedal down fully when accelerating; only shifting gears when they reach the highest possible speed; and braking abruptly. Consequently, the performance objectives for a fuel saving driving style included: (1) press down the gas pedal to 2/3 of the maximum; (2) shift gears at 15/35/55 km p/h (2000 revolutions); and (3) anticipate when there is a need to brake (i.e. less use of the brake). Next, determinants of the performance objectives were examined with a questionnaire based on the reasoned action theory. As an example, the determinants of the performance objective ‘shifting gears at 15/35/55 km p/h.’ were: attitude, self-efficacy, and habit. One related change objective was drivers explaining that shifting gears at 15/35/55 km p/h does not make the engine of the car lazy—a relevant false belief for drivers that came up in preceding qualitative research. Other change objectives were that drivers express confidence in shifting gears at 15/35/55 km p/h. or that drivers identify their own habitual patterns and list adequate coping responses. At a higher level, the management of the mail company should order cars with much more saving potential. Modeling, which involves presenting an appropriate model that performs the desired action, was one of the chosen methods. The practical application for van-drivers would be a fellow driver demonstrating the fuel saving driving style, showing less fuel use while driving the same car, over the same distance, and within the same time. The program consisted of an intervention package composed of several practical applications of theoretical methods which were combined to complement and reinforce the effect of the other applications. The theoretical methods underlying these applications were information, (physical) facilitation, model demonstrations, task assignment and control, and feedback. The information included persuasive communication about relevant beliefs and misconceptions about car engines, fuel use, and driving speed. The (physical) facilitation involved stickers on the dashboard as cues for action, and tachometers and gas flow meters for making the fuel saving behavior easier. The model demonstrations triggered interest and active learning of skills. The task assignment and control were methods at the organizational level, based on power differences: energy saving was presented as a task for the drivers, which was monitored by their local supervisor. Finally, weekly feedback on fuel consumption was provided with the aim to reinforce, monitor, and sustain performance. The program was evaluated in a field experiment. First, differences in fuel use between the experimental and control group were examined. The experimental group was found to have achieved an 8% reduction in fuel use compared to the control group. Second, reported behaviors were also compared. Van drivers in the experimental group shifted gears timely, and anticipated braking significantly more often than those in the control group.

#### Academic performance: Increasing science achievements among middle-school students

Murray et al. (2009) developed a multi-media educational program to increase science achievement among inner-city non-Asian minority middle-school students in the USA. Non-Asian minority students perform poorly and one of the reasons might be a lack of minority (Hispanic) teachers, serving as role models. Their program, HEADS-UP, was composed of select topical educational modules delivered by minority science experts. The overall program goals were increased performance (SAT10) and interest in science. Sub goals were: interest in science, confidence in ability to perform well in science, reduced fear of science, and confidence in ability to pursue science-related careers or occupations. Performance objectives were formulated for students and teachers as well as determinants, such as attitudes, perceived norms, self-efficacy, and skills. Behavior change methods targeted these determinants, and were translated into videos and classroom activities. The video stories provided vicarious learning through stories, for example modeling support from families and peers for expressing an interest in science. Other videos showed career stories of scientists, many of whom were minorities and several of whom were women. Classroom activities included worksheets, hands-on exercises, individual and group participation activities, case studies and problem-based learning. Teachers followed three training workshops. An intervention school was matched to a control school in a quasi-experimental design. Fifth-grade students were followed up for three years. At eight-grade students from the intervention school scored significantly higher on the SAT10 and reported higher interest in science. HEADS-UP is an interesting and innovative example of successfully applying Intervention Mapping in a non-health setting.

## Conclusions

Intervention Mapping (IM) is a helpful protocol for planning behavior change interventions. Most of IM applications target health behavior, but the protocol can be applied to any situation in which behavior change is desirable. IM was developed in 1998 as a reaction to a lack of comprehensive frameworks for health promotion program planning. IM is characterized by three perspectives: a social ecological approach, participation of all stakeholders, and the use of theories and evidence. IM describes the process of program planning in six steps, with each step comprising several tasks. Completion of all steps creates a blueprint for designing, implementing and evaluating an intervention based on a foundation of theoretical, empirical and practical information. Three highlights are of particular relevance for using IM: identifying the determinants of the target behavior, taking into account the theoretical parameters while applying behavior change methods, and making sure that the intervention is implemented as planned.

IM is a complex and time-consuming process, reflecting the difficulty of changing (health) behaviors. IM has been described by some authors as complex, elaborate, tiresome, expensive, and time consuming. However, the same authors have also concluded that IM assisted in bringing the development of interventions to a higher level as is evidenced by the successful applications of IM in different domains as we described above. IM helps intervention planners develop the best possible intervention.
